# Can PSMA PET detect intratumour heterogeneity in histological PSMA expression of primary prostate cancer? Analysis of [^68^Ga]Ga-PSMA-11 and [^18^F]PSMA-1007

**DOI:** 10.1007/s00259-025-07078-5

**Published:** 2025-01-17

**Authors:** Philipp Moritz Adrian Waibel, Ievgen Glavynskyi, Tobias Fechter, Michael Mix, Felix Kind, August Sigle, Cordula Annette Jilg, Christian Gratzke, Martin Werner, Oliver Schilling, Peter Bronsert, Martin Thomas Freitag, Constantinos Zamboglou, Anca-Ligia Grosu, Simon Konrad Benedikt Spohn

**Affiliations:** 1 Department of Radiation Oncology, University Medical Centre Freiburg, Robert-Koch Straße 3, 79106 Freiburg, Germany; 2 Institute for Surgical Pathology, University Medical Centre Freiburg, Freiburg, Germany; 3 Department of Nuclear Medicine, University Medical Centre Freiburg, Freiburg, Germany; 4 Department of Urology, University Medical Centre Freiburg, Freiburg, Germany; 5 German Cancer Consortium (DKTK). Partner Site Freiburg, Freiburg, Germany; 6 Core Facility Histopathology and Digital Pathology Freiburg, University Medical Centre Freiburg, Freiburg, Germany; 7 Berta-Ottenstein-Programme, Faculty of Medicine, University of Freiburg, Freiburg, Germany; 8 German Oncology Centre, European University of Cyprus, Limassol, Cyprus; 9 Biobank Comprehensive Cancer Centre Freiburg, University Medical Centre Freiburg, Freiburg, Germany

**Keywords:** Prostate cancer, Prostate-specific membrane antigen, PSMA PET/CT tracer, Histopathology, Intratumour heterogeneity

## Abstract

**Purpose:**

Prostate-specific membrane-antigen positron emission tomography (PSMA PET) is a promising candidate for non-invasive characterization of prostate cancer (PCa). This study evaluated whether PET with tracers [^68^Ga]Ga-PSMA-11 or [^18^F]PSMA-1007 is capable to depict intratumour heterogeneity of histological PSMA expression.

**Methods:**

Thirty-five patients with biopsy-proven primary PCa without evidence of metastatic disease nor prior interventions were prospectively enrolled. All patients underwent PSMA PET combined with computer tomography (CT) with either [^68^Ga]Ga-PSMA-11 (cohort I, 20 patients) or [^18^F]PSMA-1007 (cohort II, 15 patients) followed by radical prostatectomy. Specimens were scanned by ex-vivo CT and histologically prepared. On digitized whole-mount prostate sections, PCa areas with different morphologies were manually defined and H-Score of immunohistochemical PSMA expression was calculated with assistance by artificial intelligence (AI). PCa areas with similar H-Score were unified in segmentation on ex-vivo CT. After co-registration on PSMA PET-CT, Spearman’s coefficients of PSMA expression to mean and maximum standardized uptake value (SUV_mean_ and SUV_max_) were calculated. Furthermore, the agreement of the co-registered tumour areas to gross tumour volume (GTV) in PSMA PET was analysed.

**Results:**

Thirty-two patients were included in the final analysis. For histological PCa areas, immunohistochemical PSMA expression correlated significantly to SUV_mean_ and SUV_max_ (*p* < 0.001, *p* = 0.001). An approximate linear correlation between H-Score and SUV_mean_ / SUV_max_ was found for tumour areas larger than 400 μm² in histology (*p* < 0.001). Tumour areas with strong PSMA expression showed a significantly larger overlap to GTV in PSMA PET after co-registration than tumour areas with very low PSMA expression (*p* < 0.01). No significant differences were found between the two tracer cohorts (*p* = 0.72).

**Conclusion:**

PSMA PET with both [^68^Ga]Ga-PSMA-11 or [^18^F]PSMA-1007 is able to detect changes in histological PSMA expression within PCa lesions allowing biologically targeted radiotherapy.

**Supplementary Information:**

The online version contains supplementary material available at 10.1007/s00259-025-07078-5.

## Introduction

Histopathological examination, particularly the classification of the tumour morphology with the Gleason Score (GS), is currently a main factor in risk stratification of prostate cancer (PCa) and therefore decisive for the choice of therapy. At initial diagnosis, grading usually relies on tissue obtained through punch biopsies. However, PCa typically exhibits strong intratumour heterogeneity [1] which is crucial for progression, risk of metastasis and resistance to therapy [2]. Consequently, biopsies are associated with the risk of underestimating the aggressiveness of PCa: The rate of upgrading of the initial GS derived from biopsies after radical prostatectomy (RP) ranges between around 40% [3, 4] and 70% [5]. Furthermore, biopsies are an invasive procedure with risks of complications such as infection requiring hospitalization in up to 6.3% of the patients [6]. A non-invasive method for PCa grading with sufficiently high resolution therefore bears the potential of substantially improving the accuracy of risk stratification of PCa while reducing the risk of complications.

Multiparametric magnetic resonance imaging (mpMRI) is established as the gold standard in detection and evaluation of local spread of PCa and biopsy guidance [7, 8]. GS prediction based on radiomic features from mpMRI has shown promising results [9, 10], but still lacks standardization and validation to be introduced into clinical routine [7, 11]. More importantly, mpMRI has been shown to underestimate true tumour mass and miss clinically significant lesions [12, 13].

With its high sensitivity and specificity in PCa lesion detection [14, 15], positron emission tomography (PET) targeting prostate specific membrane antigen (PSMA) has gained an important role in PCa staging and treatment planning [16–19] and has shown large potential for non-invasive characterization of PCa [11, 20]. Among the tracers most frequently used for PSMA PET are [^68^Ga]Ga-PSMA-11 and [^18^F]PSMA-1007 [21]. Potential advantages of [^18^F]PSMA-1007 over [^68^Ga]Ga-PSMA-11 are the possibility of large-volume production using cyclotrons, the longer half-life and the lower positron energy, which reduces the positron range and theoretically improves spatial resolution [22]. Due to the lower urinary excretion, [^18^F]PSMA-1007 could be superior in locoregional staging [22, 23].

For both tracers [^68^Ga]Ga-PSMA-11 and [^18^F]PSMA-1007, individual studies were able to demonstrate a significant correlation between histological PSMA expression and SUV_max_ in PSMA PET of PCa lesions [24–26]. Histological expression of prostate specific membrane antigen (PSMA) is in turn correlated to GS, risk of metastasis, recurrence and castration resistance [27, 28]. However, it remained unclear whether it is possible to detect the heterogeneity of histological PSMA expression within a PCa lesion using PSMA PET.

The aim of this study was to investigate the correlation between histological PSMA expression, quantified in H-Score, and SUV_mean_ as well as SUV_max_ of [^68^Ga]Ga-PSMA-11 and [^18^F]PSMA-1007 PET in co-registered histologically defined PCa areas. An established workflow for co-registration of histological sections via ex vivo and in vivo CT with PSMA PET [29] was further developed to convey information about histological PSMA expression more precisely.

## Materials and methods

### Study design and patient characteristics

This single-centre study prospectively enrolled 35 patients with histologically confirmed prostate cancer who were scheduled for radical prostatectomy with pelvic lymphadenectomy. Histological confirmation was obtained by systematic punch biopsies with additional MR-targeted biopsies in some cases. Inclusion criteria comprised a preoperative PSMA PET-CT with no evidence of distant metastases. Patients were excluded from the study if they had undergone previous interventions such as transurethral resection of the prostate or antiandrogen therapy. All patients gave written informed consent. The study was approved by the local ethics committee (476/14 and 469/19) and conducted in accordance with the Declaration of Helsinki.

All patients underwent PET-CT with consecutively either [^68^Ga]Ga-PSMA-11 (first 20 patients, cohort I) or [^18^F]PSMA-1007 (15 patients, cohort II) followed by RP. As the histological sections were not completely available in two of the patients originally enrolled, they were excluded from further analysis. Another patient had to be excluded because the time span between PSMA PET-CT and RP was 1383 days, which was deemed too long for proper correlation.

### PET/CT Imaging and contouring of PSMA PET GTV

[^68^Ga]Ga-PSMA-11 and [^18^F]PSMA-1007 were synthesized as described in [30, 31], respectively. The patients were asked to void immediately before the PET scan. The activity of the injected [^68^Ga]Ga-PSMA-11 (cohort I) and [^18^F]PSMA-1007 (cohort II) ranged between 114 and 251 MBq and 222–370 MBq with a median of 203 MBq and 301 MBq, respectively. Whole-body PET was performed one ([^68^Ga]Ga-PSMA-11) or two ([^18^F]PSMA-1007) hours after injection. Depending on previous imaging and contraindications, contrast-enhanced CT (120 kVp, 100–400 mAs, dose modulated) or low-dose CT (120 kVp, 25 mAs) was performed for attenuation and scatter correction. Three different PET/CT devices from Philips Healthcare (USA) were used: Gemini TF TOF 64 (TF64) in 15 patients, Gemini TF Big Bore (BB) in two patients and Vereos (V) in 15 patients. According to the NU2 specification of the National Electrical Manufacturers Association (NEMA), the device resolutions were 4.8 mm (TF64/BB) and 4.2 mm (V) full-width-half-maximum in the transverse plane and 4.8 mm (TF64/BB) and 4.2 mm (V) in the axial plane [32, 33]. All systems were cross-calibrated, met the imaging guidelines of the European Association of Nuclear Medicine (EANM) and received EANM Research Ltd. (EARL) accreditations. The reconstruction methods applied are described in [20]. Reconstruction parameters for all 3 systems were adapted to have similar spatial resolution and to fulfil EARL standard 1 criteria for SUV recovery coefficients of the NEMA IEC (International Electrotechnical Commission) phantom spheres. The resulting PET images with a voxel size of 2 × 2 × 2 mm³ were normalized to standardized uptake values (SUV) in g/ml. Two radiation oncologists with at least two years of experience in interpretation of PSMA PET contoured PET “gross tumour volume” (GTV) in consensus as proposed in [34, 35] by applying a windowing of 0 to 5 SUV ([^68^Ga]Ga-PSMA-11) or 0 to 10 SUV ([^18^F]PSMA-1007) in Eclipse software (v15.1, Varian Medical Systems, USA).

### Histopathological workflow

**Fig. 1:**
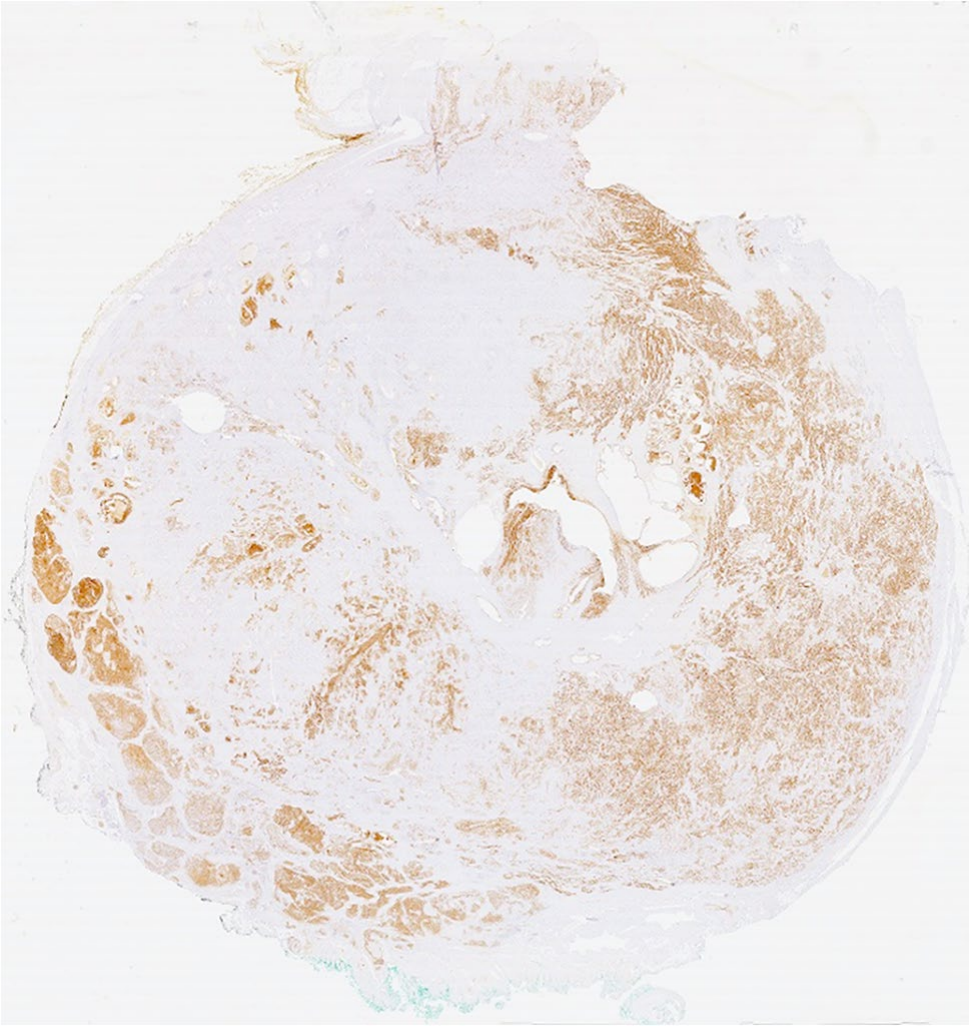
Immunohistochemical staining of PCa

Similarly to the workflow described in [36, 37], the resected and fixated prostates were embedded in agarose in a customized localizer with four-mm-spaced markers. To support orientation, the four edges of the prostates had been inked with four different colours. After an ex-vivo CT scan with the device Brilliance Big Bore (Philips Healthcare), the resected prostates were cut in the same angle as the ex-vivo CT scans perpendicular to the urethra in 4 mm wide sections. The resulting whole-mount histopathological sections were numbered from prostate basis to apex. Staining was performed with hematoxylin and eosin and ready-to-use PSMA antibodies according to routine protocols. The Ventana DP 200 slide scanner (Roche Diagnostics, Switzerland) was applied to digitize the histopathological sections with an example shown in Fig. 1. On every section, two experienced pathologists manually defined and annotated PCa areas with different morphologies (GS and growth patterns) using QuPath (v0.2.2 [38]), . For every PCa area, the surface area in µm² and the H-Score of the immunohistochemical PSMA expression were calculated with QuPath. For the semiquantitative H-Score, every cell in the PCa area was assigned a value between 0 (no PSMA expression) and 3 (strong PSMA expression). The H-Score of the specified PCa area was then calculated by multiplication of these values with the percentage share of the respective cells in the area. The H-score is therefore in the range from 0 to 300 [39].

### Co-registration and definition of tumour volumes and areas

**Fig. 2:**
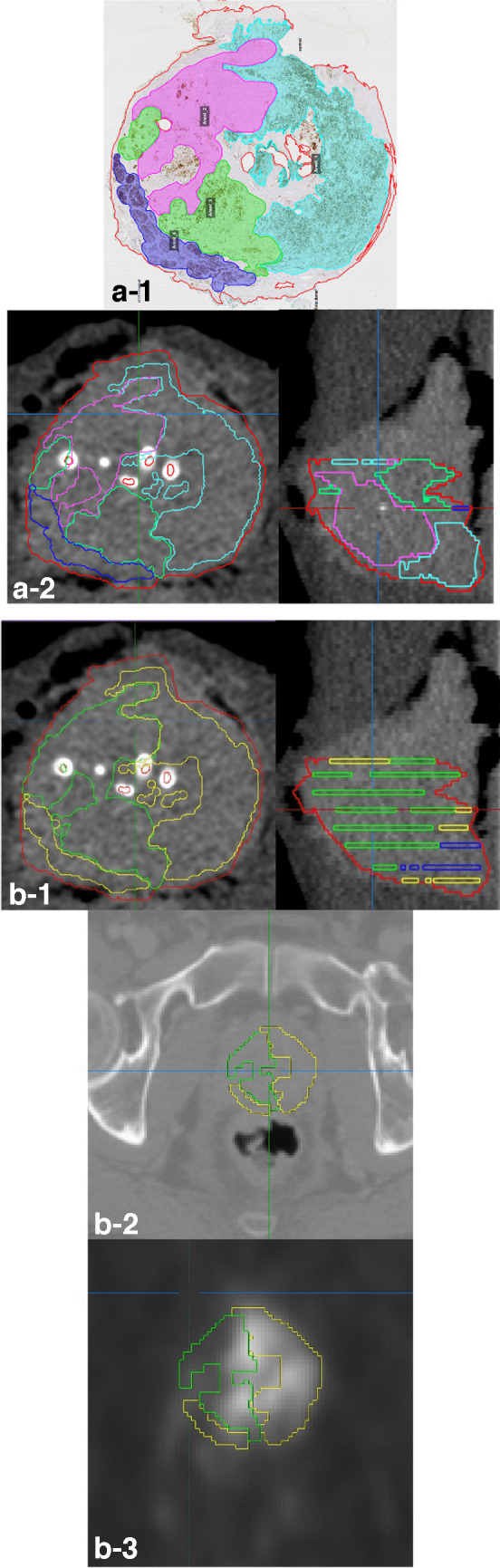
Co-registration workflow between histopathological sections and PSMA PET in MITK. In the segmentations on ex-vivo-CT, the outer contour of the whole prostate is drawn in red. **a** Segmentation on ex-vivo-CT for pathway 1. 2a-1 Digitized histopathological section with histologically defined PCa areas (histo-areas): The dark blue histo-area has been graded with a GS of 8. The other three areas have a common GS of 7b, but different histological morphology. 2a-2 Segmentation on ex-vivo-CT: Histo-areas were interpolated in direction of the z-axis (prostate base to apex) to tumour volumes. **b** Segmentation on ex-vivo-CT and co-registration with PET-CT for pathway 2. 2b-1 Histo-areas with similar PSMA expression (H-Score) were combined into one tumour area (H-Score very low – green, H-Score low – yellow, H-Score medium – blue, H-Score high – black (not shown)). 2b-2 Co-registration ex-vivo-/in-vivo-CT with tumour areas shown with green (H-Score very low) and yellow (H-Score low) contours. 2b-3 Correction of co-registration under visual comparison with PSMA PET with tumour areas shown with green (H-Score very low) and yellow (H-Score low) contours. The contours of the histo-areas on the PET-CT are shown simplified for the reader’s better understanding

The multi-step co-registration workflow between whole-mount histopathological sections and PSMA PET performed in this study is a further development of the routine established as “Manual Coregistration” in [29] using The Medical Imaging Interaction Toolkit (MITK v2015.5.2 [40]), . Its principal steps are shown in Fig. 2. First, histopathological sections were matched to ex-vivo CT by guidance of the four-mm-grid. The contours of the histologically defined PCa areas, called histo-areas in the following, were manually transferred to the corresponding ex-vivo CT slices twice applying two different segmentation routines corresponding to two different levels of resolution.

For *pathway & analysis 1*, the histo-areas were interpolated to longitudinal *tumour volumes* stretching from prostate base to apex. For *pathway & analysis 2*, the 191 histo-areas were divided into four H-Score groups according to their histological PSMA expression. The H-Score limits of these groups were selected in such a way that the groups contain a similar number of histo-areas: “H-Score very low” (H-Score 0–75, 40 areas), “H-Score low” (H-Score 76–132, 48 areas), “H-Score medium” (H-Score 133–180, 51 areas) and “H-Score high“ (H-Score 181–300, 52 areas). For each patient, the histo-areas in the same H-Score group, therefore with similar PSMA expression, were combined into one area, while no volume interpolation was performed. For distinction, these combined histo-areas are referred to as *tumour areas* in this publication.

Secondly, ex-vivo-CT was carefully matched manually to in-vivo CT including non-rigid deformations to account for ex-vivo changes. Misalignment between in-vivo CT and PET was corrected by an experienced medical physicist. Finally, to correct minor errors in the co-registration workflow, small rotations or translations of the co-registered tumour areas and volumes were performed under visual comparison with the PET images (in the range of 1 to 16 mm).

### Statistical analysis

Statistical analysis was performed in R (Version 4.3.1, R Foundation for Statistical Computing, Vienna, 2023) and Python (Version 3.11.2, Python Software Foundation). The significance level for p-values and confidence intervals (CI) was 0.05 and 0.95, respectively.

1. Tumour volumes For each tumour volume, the H-Score of histological PSMA expression was calculated by taking the area-weighted mean of the H-Scores of the included histological PCa areas (histo-areas). After co-registration, SUVmean, SUVmax and in-vivo volume were computed with a customized Python program to avoid re-sampling and the associated loss of small tumour volumes. Correlation of SUVmean and SUVmax to H-Score of histological PSMA expression was tested by calculating the Spearman rank correlation coefficients.
2. Tumour areas: Computation of H-Score and PET metrics was performed analogously. The H-Score of histological PSMA expression was calculated by taking the area-weighted mean of the H-Scores of the corresponding histo-areas. To obtain a measure for the loss of accuracy regarding the histological PSMA expression caused by the combination of histo-areas to tumour areas, the area-weighted standard deviation of the H-Score of each tumour area was calculated. The Python program already mentioned above was applied again to compute SUVmean and SUVmax of the co-registered tumour areas. The co-registered tumour areas were further compared to GTV in PSMA PET: Similarly to the Sørensen-Dice coefficient (DSC), the proportion of each tumour area that matched the GTV in PSMA PET was calculated, named GTV agreement in the following. Correlation of the resulting GTV agreement values, SUVmean and SUVmax to H-Score of histological PSMA expression was tested by computing the Spearman rank correlation coefficients. Kruskal-Wallis and Dunn’s test were applied to test for significant differences between the GTV agreement values, SUVmean and SUVmax of tumour areas of different H-Score groups and the two patient cohorts.

## Results

The characteristics of the 18 patients in cohort I ([^68^Ga]Ga-PSMA-11) and the 14 patients in cohort II ([^18^F]PSMA-1007) are summarized in Table 1. The difference of GTV in PSMA PET (ml) between the two cohorts was not significant in the Kruskal-Wallis test (*p* = 0.4). The detailed patient characteristics are to be found in the Supplementary Tables 3 and 4.

**Table 1:** Patient characteristics. GS and TNM are based on whole-mount histopathological sections from radical prostatectomy

	Cohort I	Cohort II
	Median (Range)	
Age (y)	62 (51–74)	70 (53–80)
PSA at imaging (ng/ml)	16,9 (6,1–218,0)	17,4 (4,3–108,0)
Time between PET and RP (d)	29 (1–159)	25 (1–43)
GTV in PSMA PET (ml)	10,3 (0,8–87,7)	5,5 (0,6–48,6)
	Frequency (%)	
pT2a	0	21
pT2c	39	14
pT3a	28	36
pT3b	33	29
pN0	61	86
pN1	39	14
GS 7a	39	0
GS 7b	28	50
GS 8	22	21
GS 9	11	29

The histopathological workup of resected prostates from these 32 patients resulted in 191 histologically defined PCa areas (histo-areas) with a median area of 60 μm² (interquartile range (IQR) 23–222 μm²). Starting from these histo-areas, 56 tumour volumes and 76 tumour areas were successfully co-registered and analyzed as described in 2.4 and 2.5.

1. Tumour volumes: The co-registered tumour volumes had a median in-vivo volume of 0.34 ml (IQR 0.10–1.93 ml). The mean H-Score was 132.2. The overall medians of SUVmean and SUVmax were 5.5 g/ml (IQR 3.3–10.8 g/ml) and 10.9 g/ml (IQR 6.0–24.1 g/ml), respectively. A significant and similar correlation was found between H-Score of histological PSMA expression and both SUVmean (Spearman’s rho ρ = 0.36, *p* < 0.01, CI [0.11, 0.57]) and SUVmax (ρ = 0.40, *p* < 0.01 CI [0.16, 0.60]). In the 16 co-registered tumour volumes with an in-vivo volume larger than 1 ml, correlation of H-Score to SUVmean was significantly stronger: ρ = 0.88 (*p* < 10^−15^, CI [0.68, 0.96]).
2. Tumour areas: The median histological area of the tumour areas was 93 μm² (IQR 41–232 μm²). The tumour areas had a mean H-Score of 137.6 with a mean standard deviation of only 4.7. As already described in [41], the median SUVmean and SUVmax of the tumour areas from cohort I (tracer [68Ga]Ga-PSMA-11) was significantly lower than the median SUVmean and SUVmax of the tumour areas from cohort II (tracer [18F]PSMA-1007): 3.81 g/ml and 6.81 g/ml compared to 7.32 g/ml and 14.19 g/ml (*p* < 0.01). Median agreement of the co-registered tumour areas with GTV in PSMA PET was 60% (IQR 37–90%), 66% (IQR 41–88%) for cohort I and 58% (IQR 37–92%) for cohort II, with no significant differences between the two tracer cohorts (*p* = 0.72).

**Fig. 3:**
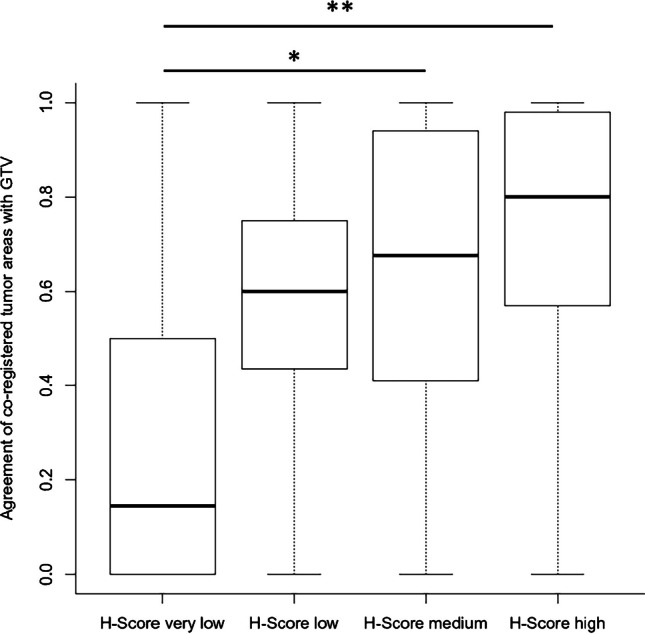
Relative agreement of co-registered tumour areas with GTV in PSMA PET in dependence of the histological PSMA expression. The median agreement of the areas of each H-Score group was 15% (H-Score very low), 60% (H-Score low), 68% (H-Score medium) and 84% (H-Score high). The differences were significant between the group “H-Score very low” and the groups “H-Score medium” and “H-Score high” (*p* = 0.02, *p* < 0.01)

The GTV agreement correlated significantly to the H-Score of histological PSMA expression (ρ = 0.4, *p* < 0.001). As shown in Fig. 3, a significantly larger overlap to GTV in PSMA PET was observed for co-registered tumour areas with medium and high H-Scores compared to co-registered tumour areas with very low H-Scores (*p* = 0.02, *p* < 0.01).

**Fig. 4:**
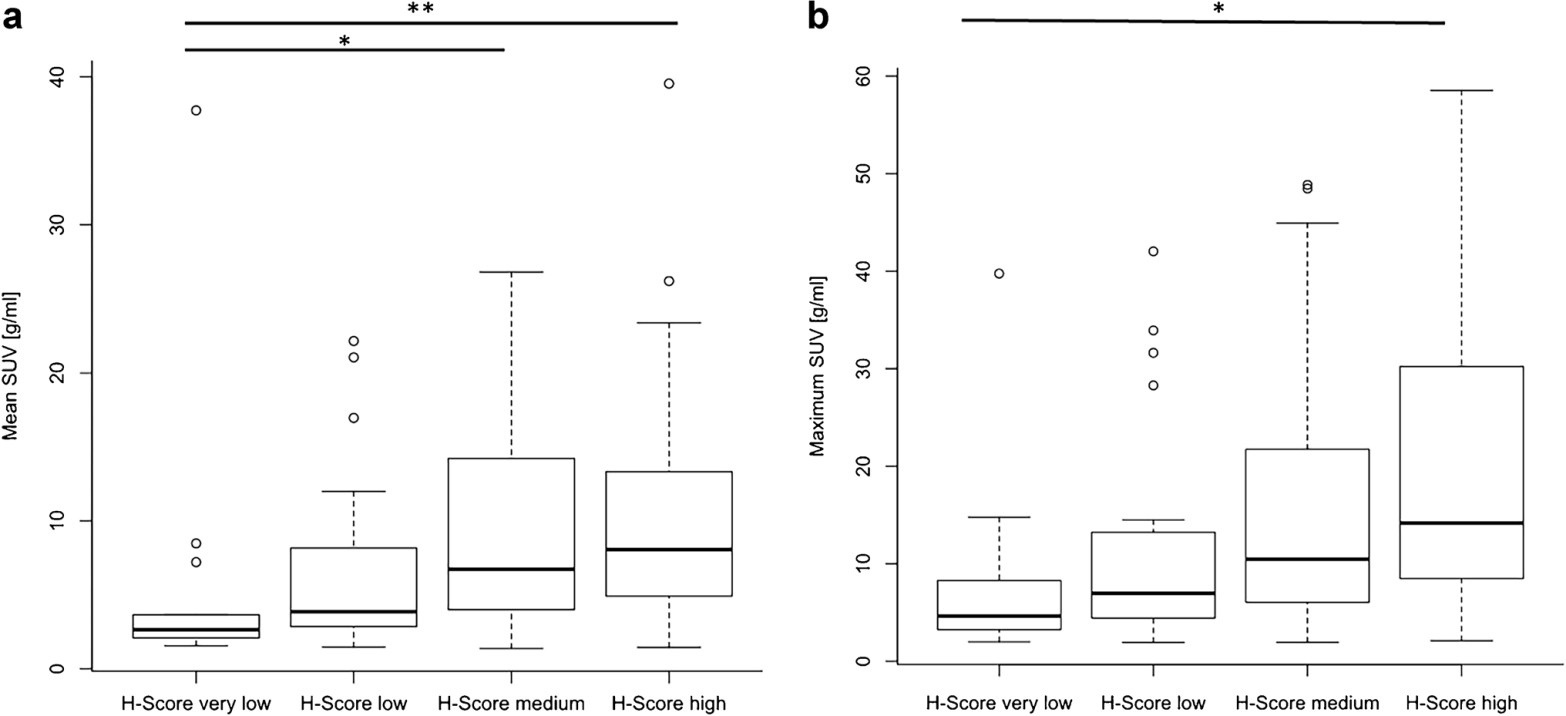
SUV_mean_ (**a**) and SUV_max_ (**b**) of co-registered tumour areas in PSMA PET in dependence of the histological PSMA expression. **a** The medians of the SUV_mean_ of the areas of each H-Score group were 2.62 (H-Score very low), 3.89 (H-Score low), 6.08 (H-Score medium) und 7.14 (H-Score high) g/ml. The differences were significant between the group “H-Score very low” and the groups “H-Score medium” and “H-Score high” (*p* = 0.02, *p* < 0.01). **b** The medians of the SUV_max_ of the areas of each H-Score group were 4.64 (H-Score very low), 6.98 (H-Score low), 10.47 (H-Score medium) und 14.19 (H-Score high) g/ml. The differences were significant between the groups “H-Score very low” and “H-Score high” (*p* = 0.01)

**Fig. 5:**
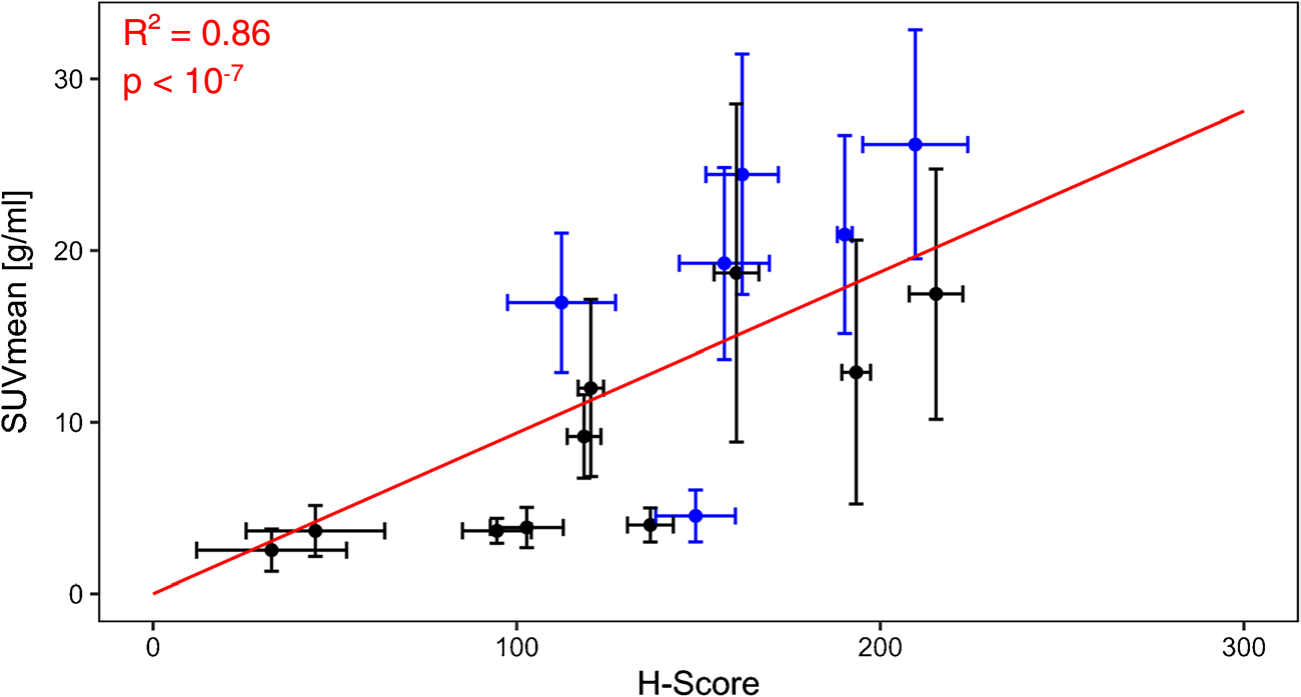
SUV_mean_vs. H-Score of tumour areas with a histological area > 400 μm². The points for the ten areas from cohort I (tracer [^68^Ga]Ga-PSMA-11) are shown in black, the points for the six areas from cohort II (tracer [^18^F]PSMA-1007) in blue. The error bars correspond to the respective standard deviations. The linear regression (red line) with a fixed y-intercept of 0 resulted in a quadratic deviation of R² = 0.86 for a slope of 0.09 ± 0.01 g/ml (*p* < 10^−7^)

**Fig. 6:**
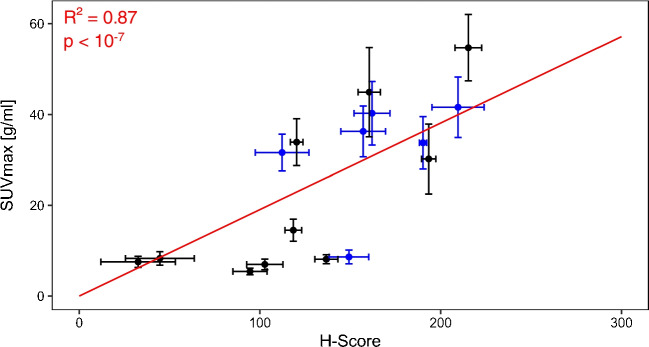
SUV_max_vs. H-Score of tumour areas with a histological area > 400 μm². The points for the ten areas from cohort I (tracer [^68^Ga]Ga-PSMA-11) are shown in black, the points for the six areas from cohort II (tracer [^18^F]PSMA-1007) in blue. The error bars correspond to the respective standard deviations. The linear regression (red line) with a fixed y-intercept of 0 resulted in a quadratic deviation of R² = 0.87 for a slope of 0.19 ± 0.02 g/ml (*p* < 10^−7^)

Figure 4 shows that tumour areas with a very low H-Score had a significantly lower SUV_mean_ and SUV_max_ than tumour areas with a high H-Score. As shown in detail in Table 2, the Spearman correlation analysis for the co-registered tumour areas yielded a significant correlation between the H-Score of histological PSMA expression and SUV_mean_ as well as SUV_max_. The correlation was significant for the tumour areas of both tracer cohorts separately with no significant differences between the correlation coefficients. Correlation of H-Score to SUV_mean_ was significantly stronger in the 16 co-registered tumour areas with a histological area over 400 μm². For these tumour areas, an almost linear correlation of H-Score to SUV_mean_ and SUV_max_ was found (Figs. 5 and 6). A correlation analysis including only the tumour areas of the patients with a PSMA PET GTV larger than 5 ml resulted in only a slight increase in the correlation coefficients.

**Table 2:** Analysis 2 (tumour areas): Spearman’s rank correlation coefficients ***ρ ***of histological PSMA expression (H-Score) to SUV_mean _or SUV_max _in PSMA PET

	Variable 2	ρ	*p* value	95% confidence interval
All areas	SUV_mean_	**0.39**	< 0.001	[0.18, 0.56]
All areas	SUV_max_	**0.36**	0.001	[0.15, 0.54]
Areas cohort I	SUV_mean_	**0.37**	0.01	[0.11, 0.62]
Areas cohort II	SUV_mean_	**0.37**	0.03	[0.04, 0.64]
Areas cohort I	SUV_max_	**0.30**	0.05	[0.004, 0.55]
Areas cohort II	SUV_max_	**0.37**	0.03	[0.04, 0.64]
Areas > 400 μm²	SUV_mean_	**0.83**	< 10^−4^	[0.57, 0.94]
Areas > 400 μm²	SUV_max_	**0.79**	< 0.001	[0.47, 0.92]
Areas cohort I > 400 μm²	SUV_mean_	**0.93**	< 0.01	[0.58, 0.99]
Areas cohort II > 400 μm²	SUV_mean_	**0.89**	0.03	[0.26, 0.99]
Areas | GTV > 5 ml	SUV_mean_	**0.41**	< 0.01	[0.15, 0.62]
Areas | GTV > 5 ml	SUV_max_	**0.38**	< 0.01	[0.11, 0.60]

The patients were divided in two cohorts based on the PSMA-PET tracer used: [^68^Ga]Ga-PSMA-11 in cohort I and [^18^F]PSMA-1007 in cohort II. 16 out of 76 co-registered tumour areas had a histological area over 400 μm², 10 from cohort I and 6 from cohort II. 14 out of 32 patients had a GTV larger than 5 ml

## Discussion

To the best of our knowledge, this is the first study to investigate the correlation between histological PSMA expression and uptake in PSMA PET on the level of histologically defined PCa areas. In previous studies testing this correlation, histopathological sections were directly assigned to tumour lesions in PSMA PET-CT or vice versa [24–26, 42, 43]. In contrast, we performed a multi-step co-registration workflow (Fig. 2) of histological PCa areas via ex-vivo and in-vivo CT to PSMA PET. Furthermore, this study allowed for the comparison of the two tracers [^68^Ga]Ga-PSMA-11 (cohort I) and [^18^F]PSMA-1007 (cohort II) with regard to their correlation to histopathology.

Co-registration and correlation analysis was performed twice in two different levels of resolution. First, our study confirms the correlation between histological PSMA expression and the uptake in PSMA PET for co-registered tumour volumes similar to previous lesion-based analyses. However, since the histopathological examination revealed relevant changes in histological PSMA expression and morphology within these tumour volumes, a second, high-resolution analysis without volume interpolation was performed. The histologically defined PCa areas were only combined to tumour areas with similar PSMA expression quantified as H-Score. As the mean standard deviation of the H-Score of the tumour areas was merely 4.7 compared to a mean H-Score of 132.2, our analysis of tumour areas indeed corresponds to a correlation analysis at the level of histological PCa areas.

Consequently, the highly significant correlation of H-Score to SUV_mean_ and SUV_max_ observed for the co-registered tumour areas indicates that PSMA PET can detect differences in histological PSMA expression and tumour morphology not only between different PCa lesions, but even within these lesions. In addition, our study shows an equivalence between the two tracers [^68^Ga]Ga-PSMA-11 and [^18^F]PSMA-1007 with regard to the resolution of histological PSMA expression and the correlation of co-registered tumour areas to PSMA PET GTV. PET with both [^68^Ga]Ga-PSMA-11 or [^18^F]PSMA-1007 is able to depict intratumour heterogeneity in histological PSMA expression.

These results are especially relevant for the radiotherapy of PCa, as the traditional concept of irradiating the whole prostatic gland homogenously is currently challenged by treatment strategies incorporating focal dose escalation. In the FLAME trial, an increased radiation dose delivered to intraprostatic PCa lesions visible on multiparametric magnetic resonance imaging (mpMRI) resulted in a significantly improved oncologic outcome [44]. An even more ambitious approach to focal dose escalation, which goes beyond the consideration of individual tumour anatomy, is biologically targeted radiotherapy. Its objective is to administer a non-homogeneous dose in the tumour according to its spatially defined biological characteristics. In our study, tumour areas with a strong PSMA expression showed significantly greater agreement with GTV in PSMA PET compared to areas with a weak expression. Since histological PSMA expression correlates with dedifferentiation, risk of metastasis, recurrence and castration resistance [27, 28], our findings suggest that an increased radiation dose on PSMA PET GTV – as tested in the currently ongoing HypoFocalSBRT study [18] - might further improve therapeutic outcomes. More refined concepts of biologically targeted radiotherapy with, for example, a multi-stage dose concept depending on SUV in PSMA PET could be tested in future studies.

However, further fundamental research is needed to integrate important current findings into the understanding of PCa biology. Overall, the study by Heetman et al. published last year confirmed the correlation of Gleason score to histological PSMA expression and maximum SUV in PCa [26]. At the other hand, it showed that in some cases, the dedifferentiation of PCa is accompanied by a loss of PSMA receptors. PCa with Gleason score 9 showed a lower mean histological PSMA expression and a lower maximum SUV than PCa with Gleason score 8 [26]. Likewise, the implications of the recently observed increased radioresistance of PCa lesions with low SUV_max_ and SUV_mean_ in PSMA PET need to be further explored [45].

In our study, the correlation between histological PSMA expression and the uptake in PSMA PET was dependent on the size of the analysed tumour volumes and areas: Spearman’s coefficients of H-Score to SUV_mean_ and SUV_max_ were clearly, in part significantly, higher for the tumour volumes with an in-vivo volume over 1 ml and the tumour areas with a histological area over 400 μm². A possible explanation for this size dependency of the correlation is the partial volume effect (PVE): It causes small tumours with a high tracer uptake to appear larger and less bright in PET imaging [46]. Since the initial total tumour volume was not collected in this study, we used the PET-GTV already biased by the PVE as a rough estimate. According to measurements of Alginate spheres with known volume and tracer uptake with the PET devices used [47], the PVE could be relevant for the tumours of 14 of the 32 patients with a GTV smaller than 5 ml. However, sorting out the areas of these patients resulted in only a slight increase in the correlation coefficients. The PVE therefore does not appear to be the sole explanation for the size dependence of the correlation. Tumour volume measurement in MRI could enable a more precise assessment and a possible correction of PVE.

A second explanation for the size dependency for the correlation between H-Score and SUV are minor errors in the co-registration. These are less significant with a larger tumour area or a larger tumour volume due to the larger sample size. Matching of ex-vivo CT to in-vivo CT appears to be particularly error-prone, as the shape of the prostate differs significantly: in vivo, it is deformed by its neighbouring organs, the bladder and rectum, while the loss of blood volume and histopathological fixation triggers an inhomogeneous shrinkage process ex vivo. The PET-based position correction of the co-registered areas and lesions might not have been able to compensate completely for the resulting inaccuracies.

A fundamental limitation for the correlation between histological PSMA expression and uptake in PSMA PET are the different experimental conditions. Immunohistochemical detection of PSMA takes place almost directly on the tumour cells that have been removed from the body. PSMA PET tracers must bind to the tumour cells within the living body requiringsufficient vascularization of all parts of the tumour. Information from MRI perfusion measurements could therefore be included in future studies.

Further limitations of this study are the single centre design and the limited number of patients, which precluded a sensitivity analysis after removing outliers. No correction was made for multiple testing. Finally, unlike the SUV, the H-Score is only a semi-quantitative variable as the expression level of individual cells is graded in discrete levels. We would like to emphasize that the quantification of histological PSMA expression with H-Score was carried out semi-automatically with artificial intelligence (AI) assistance in QuPath and thus more reproducible than in most previous studies testing the correlation of uptake in PSMA PET [24–26, 42].

This study underlines the potential of PSMA PET for the non-invasive characterization of PCa. Since the results represent a successful positive control of the refined co-registration workflow, it could be used for further high-resolution analyses of the correlation of uptake in PSMA PET signal with clinically relevant histopathological features such as ALDH1A1 expression. Furthermore, the analysis of radiomic features of PSMA PET could be of considerable benefit for the non-invasive characterization and risk stratification of PCa. However, further research into reproducibility and standardization is required [11, 48, 49].

## Supplementary information

Below is the link to the electronic supplementary material.

ESM 1 (DOCX 20.6 KB)
