# Supplementary material for: Can PSMA PET detect intratumour heterogeneity in histological PSMA expression of primary prostate cancer? Analysis of [68Ga]Ga-PSMA-11 and [18F]PSMA-1007
Source: Eur J Nucl Med Mol Imaging. 2025 Jan 17;52(6):2023–33. doi: 10.1007/s00259-025-07078-5 (PMC12014795; doi:10.1007/s00259-025-07078-5)
Supplement: Supplementary file 1 — (DOCX 20.6 KB) [file 259_2025_7078_MOESM1_ESM.docx]

**Table 3: Characteristics of patients in cohort I (PSMA PET tracer [^68^Ga]Ga-PSMA-11)** GS was based on whole-mount histopathological sections from radical prostatectomy. GTV in PSMA PET was contoured by two experienced radiation oncologists in consensus by applying a windowing of 0 to 5 SUV.

| Patient | Age (y) | PSA at imaging (ng/ml) | #Days between PET  and RP | TNM | GS | PSMA PET GTV (ml) |
| --- | --- | --- | --- | --- | --- | --- |
| 1 | 67 | 6.1 | 17 | pT3a pN1 cM0 | 7a | 13.1 |
| 2 | 61 | 10.6 | 1 | pT2c pN0 cM0 | 7a | 5.1 |
| 3 | 52 | 51.1 | 50 | pT3b pN1 cM0 | 9 | 24.4 |
| 4 | 60 | 49.0 | 19 | pT2c pN1 cM0 | 7a | 4.8 |
| 5 | 73 | 25.5 | 19 | pT2c pN0 cM0 | 7a | 3.1 |
| 6 | 59 | 9.2 | 22 | pT2c pN0 cM0 | 7b | 3.5 |
| 7 | 74 | 8.8 | 85 | pT2c pN0 cM0 | 7a | 0.8 |
| 8 | 74 | 15.0 (14.0 at RP) | 159 | pT2c pN0 cM0 | 7a | 1.4 |
| 9 | 62 | 47.2 | 26 | pT3b pN1 cM0 | 8 | 46.6 |
| 11 | 68 | 11.0 | 48 | pT3a pN0 cM0 | 7a | 3.0 |
| 12 | 51 | 17.4 | 6 | pT3a pN0 cM0 | 7b | 11.4 |
| 13 | 48 | 23.0 | 36 | pT3b pN1 cM0 | 7b | 22.1 |
| 14 | 76 | 20.7 | 40 | pT2c pN0 cM0 | 7b | 14.9 |
| 15 | 59 | 15.8 | 39 | pT3b pN1 cM0 | 9 | 28.8 |
| 17 | 53 | 16.3 | 20 | pT3a pN0 cM0 | 8 | 9.1 |
| 18 | 72 | 28.9 | 43 | pT3b pN1 cM0 | 8 | 25.8 |
| 19 | 70 | 16.0 | 31 | pT3a pN0 cM0 | 7b | 3.0 |
| 20 | 67 | 218.0 | 18 | pT3b pN0 cM0 | 8 | 87.7 |

**Table 4: Characteristics of patients in cohort II (PSMA PET tracer [^18^F]PSMA-1007)** GS was based on whole-mount histopathological sections from radical prostatectomy. GTV in PSMA PET was contoured by two experienced radiation oncologists in consensus by applying a windowing of 0 to 10 SUV.

| Patient | Age (y) | PSA at imaging (ng/ml) | #Days between PET  and RP | TNM | GS | PSMA PET GTV (ml) |
| --- | --- | --- | --- | --- | --- | --- |
| 21 | 70 | 4.3 | 6 | pT2a pN0 cM0 | 7b | 1.4 |
| 22 | 66 | 17.2 | 1 | pT3a pN0 cM0 | 7b | 4.7 |
| 23 | 70 | 61.0 | 23 | pT3b pN0 cM0 | 7b | 10.8 |
| 24 | 69 | 103.0 | 11 | pT3a pN0 cM0 | 9 | 15.9 |
| 25 | 76 | 5.0 | 5 | pT2c pN0 cM0 | 7b | 2.8 |
| 26 | 80 | 8.6 | 34 | pT2a pN0 cM0 | 9 | 3.7 |
| 28 | 53 | 72 | 43 | pT3b pN1 cM0 | 9 | 48.6 |
| 29 | 64 | 19.5 | 6 | pT3a pN0 cM0 | 7b | 6.1 |
| 30 | 72 | 24.8 | 20 | pT2a pN0 cM0 | 8 | 0.6 |
| 31 | 74 | 13.9 | 27 | pT3a pN0 cM0 | 7b | 1.5 |
| 32 | 66 | 17.5 | 31 | pT3b pN1 cM0 | 9 | 38.8 |
| 33 | 72 | 22.0 | 34 | pT2c pN0 cM0 | 7b | 8.3 |
| 34 | 73 | 5.0 | 35 | pT3b pN0 cM0 | 8 | 12.5 |
| 35 | 67 | 6.4 | 34 | pT3a pN0 cM0 | 8 | 4.8 |
